# Consultation-Liaison Psychiatry Services in Ireland: A National Cross-Sectional Study

**DOI:** 10.3389/fpsyt.2021.748224

**Published:** 2021-11-29

**Authors:** Anne M. Doherty, Rosie Plunkett, Katherine McEvoy, Eric Kelleher, Maurice Clancy, Elizabeth Barrett, Elaine Greene, Eugene Cassidy, William Lee, Siobhan MacHale

**Affiliations:** ^1^University College Dublin, Dublin, Ireland; ^2^Mater Misericordiae University Hospital, Dublin, Ireland; ^3^Holles Street Hospital, Dublin, Ireland; ^4^University Hospital Galway, Galway, Ireland; ^5^Cork University Hospital—CUH, Cork, Ireland; ^6^University College Cork, Cork, Ireland; ^7^University Hospital Waterford, Waterford, Ireland; ^8^Temple Street Children's University Hospital, Dublin, Ireland; ^9^St. James's Hospital, Dublin, Ireland; ^10^University of Exeter, Exeter, United Kingdom; ^11^Beaumont Hospital, Dublin, Ireland

**Keywords:** consultation-liaison (C-L) psychiatry, health services research [MeSH], hospital psychiatry, liaison psychiatry, mental health, Ireland

## Abstract

**Objective:** This study aimed to describe the provision of consultation-liaison psychiatry (CLP, also known as liaison psychiatry) services in acute hospitals in Ireland, and to measure it against recommended resourcing levels.

**Methods:** This is a survey of all acute hospitals in Ireland with Emergency Departments, via an electronic survey sent by email and followed up by telephone calls for missing data. Data were collected on service configuration, activity, and resourcing. Data were collected from CLP or proxy services at all acute hospitals with an Emergency Department in Ireland (*n* = 29). This study measured staffing and activity levels where available.

**Results:** None of the services met the minimum criteria set out by either national or international guidance per 500 bed general hospital.

**Conclusions:** CLP is a relatively new specialty in Ireland, but there are clear international guidelines about the staffing levels required to run these services safely and effectively. In Ireland, despite clear national guidance on staffing levels, no services are staffed to the levels suggested as the minimum. It is likely that patients in Ireland's acute hospitals have worse outcomes, and hospitals have unnecessary costs, due to this lack. This is the first study of CLP provision in Ireland and demonstrates the resource constraints under which most services work and the heterogeneity of services nationally.

## Introduction

Consultation-Liaison Psychiatry (CLP), also known as liaison psychiatry is a subspecialty of adult psychiatry and refers to clinical services which deliver care at the intersection of mental and physical health care. CLP provides specialist medical expertise of the management of conditions which occur in areas overlapping mental and physical healthcare. Internationally, this specialty is known variously as liaison psychiatry, psychological medicine, and general hospital psychiatry, (1). It is delivered in general or acute hospital settings. A key component of the work of CLP teams is to the Emergency Departments (ED) of their hospitals. The service provided to people who present following self-harm in Ireland is guided by the National Guidelines of the National Clinical Programme for people presenting to EDs following self-harm (NCP-SH), which in addition to providing mental health nursing staffing for EDs, also has guidelines on evidence-based practise such as full biopsychosocial assessments for all people who present, the co-production of an emergency care plan and communication with and bridging to next care (2). Some services provide outpatient services, which accept referrals from medical and surgical clinics in secondary care. Where a hospital has supra-regional or national programmes which require dedicated psychiatry resources for optimal patient care, such as organ transplantation programmes, neurological services or haematology-oncology hubs, there are often additional CLP resources to support the associated additional complex specialist mental health need associated with these.

Although CLP is a relatively new specialty, there has been much development in the past decade of service-based research which has aimed to quantify the activity and best practise of CLP services and to define needs and future development. The seminal publication in 2011 of the economic evaluation of the Rapid Assessment and Interface Discharge (RAID) service in Birmingham was pivotal in drawing the attention of funders of healthcare services in the UK to the potential of CLP services to effect cost savings. This study reported that every £1 invested in CLP services would effect a saving of £4 for that hospital (3, 4). Since this initial publication there have been further publications replicating RAID in examining the impact of CLP on the economics of the hospitals in which they are based (5–7). The effectiveness of CLP services has been supported by a systematic review conducted by Wood et al. and a narrative review conducted by the Netherlands Psychiatric Association conducted as part of the development of their guidelines (8, 9).

In England, near-annual surveys have examined the CLP services provided at all acute hospitals with EDs (10). These are in the context of commissioning guides which examined the key factors which influence the success of CLP services, set standards for good services, and described and named differing levels of service provision (11). There are also specific government targets for growth in the specialty in England [5 year forward View in Mental Health].

In Ireland, the document a vision for change provided clear guidance on staffing levels for all mental health services (12). Although this document was published 15 years ago and refreshed in 2020, there has not been any assessment of the degree to which CLP services are resourced to the minimum levels of this standard.

In this study we aimed to examine the nature of CLP services in Ireland and to define the key components of the psychiatry service provision at acute hospitals in Ireland to inform future work.

## Methods

### Setting and Sample

The sample consisted of all acute hospitals in Ireland with an ED (Model 4 and Model 3 hospitals—see below) in 2020, and were identified from the Health Service Executive website (https://www.hse.ie/eng/services/list/3/acutehospitals/hospitalgroups.html) and the National Clinical Programme for Self Harm. A Model 4 hospital provides 24/7 ED, acute surgery, acute medicine, critical care, tertiary care and, in many instances, supra-regional care. A Model 3 hospital has a 24/7 ED and provides acute medical and surgical care, and is equivalent to a district general hospital.

At each hospital site, we identified which components of CLP services were available typically defined by the part of the hospital covered by that component—for example: ED, ward referrals, links to specialist services, outpatient clinics.

We aimed to identify the keys component of the service and associated characteristics such as staff mix, working hours, patient groups seen.

### Design

This was a cross-sectional electronic survey disseminated by email and text messaging with follow up telephone interviews where required. All hospitals in Ireland with an Emergency Department (ED) were included. As there is a small body of consultants in CLP in Ireland, this was disseminated by email and text message.

In hospitals where there was no consultant the research team made contact with the CLP or proxy service by telephoning the hospital and seeking a clinician in the service. The survey was conducted via the online survey, or by telephone.

### Measures

The survey ran from October to November 2020 for Model 4 hospitals, and from March to April 2021 for Model 3 hospitals. The survey was brief and allowed flexible (free text) responses. Response was by email or telephone. Non-responding hospital sites were followed up by email and telephone.

The primary outcome measure was the level of service provision as set out in A Vision for Change, and each service was benchmarked against this standard.

This paper also compared services against international benchmarking, mainly the English publications in this area. Consistent with the work of the LP-MAESTRO study of Walker et al. we used the same variables derived from the UK based gold-standard RAID services. “Original RAID” (variable 1) is based on the description provided in Tadros et al. of the original RAID service evaluated at Birmingham City Hospital (4); “modified RAID” (variable 2), is based on the profile of current services in Birmingham still known as RAID. Each service was characterised by whether or not the met the criteria for the two RAID variables. In addition, responses on staffing level and scope of work were used to categorised each service according to recent guidance from NHS England that was created to help commissioners in planning service delivery: Comprehensive (full liaison provision), Enhanced 24 (staffed according to the original RAID model), Core 24 (provides acute provision for a hospital with an ED, but no outpatient work) and Core (intended for less busy hospitals); and services not meeting Core criteria were classified as subCore (see Table 1) (11, 13).

**Table 1:** Comparison of different models of Consultation Liaison Psychiatry provision—NHS-NICE, RAID & RANZCP CLP Model for Victoria, Australia.

	**NHS-NICE**				**RAID**		**RANZCP CLP Model for Victoria, Australia** **(“Baseline” service for admitted patients only–not ED)**
	**Core**	**Core 24**	**Enhanced**	**Comprehensive**	**Original**	**Modified**	
Hours of service	Working hours	24/7	24/7	24/7	24/7	24/7	24/7
Age groups	Over 16	Separate older adult team	Separate older adult team	Separate older adult team	All age team	All age team	All ages
Response targets—ED	n/a	n/a	n/a	n/a	1 h	1 h	n/a
Response targets—wards	n/a	n/a	n/a	n/a	24 h	24 h	80% in 24 h
Including self-harm	Yes	Yes	Yes	Yes	Yes	Some	Yes
Out-patients	No	No	Yes	Yes	Yes	Some	No
**Staffing**							
Psychiatrists							
*Consultant*	2	2	2	5			2
*Non-consultant hospital doctors (including trainees)*	2	2	4	2			3.5
Nursing	8	13	10	29			2.5
Psychology/other therapists	0	4	2	16			1
Manager	1.2	1.2	1.2	4			1
Administrator (incl. business support)	2.6	3	3	13			1

*RAID and RANZCP CLP Model for Victoria, Australia*.

### Patient and Participant Involvement

Further work will focus on patient experience and incorporate the patient voice into future developments. This study was however a more basic picture of resourcing, services provided and activity.

### Statistics

Data were entered into SPSS for statistical analysis. Given the heterogeneity of the services examined and the lack of hypothesis, descriptive statistics were generated, but there was no role for more complex statistical analysis.

## Results

Data were collected from 100% (*n* = 29) of clinical sites where there is an emergency department: nine Model 4 hospitals, 18 Model 3 hospitals and 2 tertiary paediatric hospitals. Each service was benchmarked against the levels of service provision as set out in A Vision for Change. Of the 9 Model 4 hospitals, 5 (56%) are located in Dublin along with both paediatric hospitals. The remaining Model 4 hospitals are based in the other urban centres (Cork, Limerick, Galway and Waterford). Of the 18 Model 3 hospitals, 17 (94%) are located outside the capital, Dublin.

### Staffing

#### Benchmarking Against Minimum Standards, and Classification

No services met the minimum level of staffing as per A Vision for Change, either the original or modified RAID services or any of the levels of the NHS England/ NICE recommendations—all were “sub core” as per the NHS/NICE standards (Table 2) (13). Model 4 hospitals had double the number of beds of Model 3 hospitals with significantly higher activity in the ED setting, but more significantly in terms of patients seen, both as inpatients and outpatients.

**Table 2:** Characteristics of Model 3, Model 4, & Paediatric hospitals in Ireland, activity, and models of working.

	**Model 4 (*n* = 9)**	**Model 3 (*n* = 18)**	**Paediatric (*n* = 2)**
	**Mean (SD)**	**Mean (SD)**	**Mean (SD)**
Beds per hospital, mean (SD)	688.3 (199.1)	271.2 (105.1)	215 (190.9)
ED activity, mean (SD)^a^	1675 (282.4)	737 (324.1)	725 (388.9)
Ward activity, mean (SD)^a^	983.3 (223.6)	319 (258.1)	120 (42.4)
Self-harm referrals, mean (SD)^b^	874.8 (223.6)	375.4 (182.6)	90 (84.8)
Out-patient new activity, mean (SD)^c^	311.5 (398.8)	5.2 (14.8)	172.5 (201.5)
Out-patient return activity, mean (SD)^c^	478 (674.8)	8.9 (36.9)	1750 (2474.9)
	***n (%)***	***n (%)***	***n (%)***
Extended working hours, *n* (%) 8–8	2 (22.2)	3 (16.7)	0 (0)
>18 h/day but <24 h	1 (11)	0 (0)	0 (0)
24/7	0 (0)	0 (0)	0 (0)
Funding source, *n* (%) acute hospital only	2 (22.2)	0 (0)	1 (50)
Acute hospital with NCP-SH	4 (44.5)	0 (0)	1 (50)
Mental health service with NCP-SH	3 (33.3)	16 (88.9)	0 (0)
Hybrid	2 (22.2)	2 (11.1)	0 (0)
Assessment in ED, *n* (%) In parallel with emergency medicine	7 (77.8)	15 (83.3)	1 (50)
Directly from triage in general	3 (33.3)	9 (50)	0 (0)
Directly from triage on occasion	2 (22.2)	1 (5.6)	0 (0)
Adequate office space, *n* (%)	2 (22.2)	14 (77.8)	2 (100)
Office in acute hospital, *n* (%)	8 (88.9)	10 (55.6)	2 (100)

a *For Model 3, n = 12; Model 4, n = 5*.

b *For Model 3, n = 11; Model 4, n = 5*.

c *For Model 3 n = 2 (the remainder do not have OPD), for Model 4, n = 5 (no OPD n = 2, data unavailable n = 2)*.

*NCP-SH, Funding from the National Clinical Programme for people presenting to the ED following Self-Harm*.

No service met the staffing requirements set out in Australia for Victoria by the Royal Australian & New Zealand College of Psychiatrists (14). No services operated 24/7: the majority of sites out of hours just an on-call junior doctor on site who can call an off-site consultant for advice.

#### Model 4 Hospitals

The staffing levels of Model 4 hospitals are outlined in Table 3. The mean number of consultants is 1.6 WTE (SD 0.7) and of registrars or junior doctors is 3 WTE (SD 1.9). Seven of the 9 hospitals (78%) had at least 1 WTE consultant /500 beds, and seven had at least 1 registrar or junior doctor per 500 beds.

**Table 3:** Staffing at Model 3, Model 4 & Paediatric hospitals in Ireland in absolute numbers and per 500 beds.

	**WTE /hospital**	**WTE/hospital**	**WTE/500 beds**	**Number meeting minimum standard (WTE required for compliance with AVFC/500 beds)**
	**Median (range)**	**Mean (SD)**	**Mean (SD)**	
**Model 4 hospitals** (***n*** **=** **9**)				
Clinical nurse specialist (total)	4 (3–6)	3.8 (1.3)	2.9 (0.9)	0 (5)
Clinical nurse specialist (National clinical programme, self-harm)	2 (0–3)	1.3 (1.3)	0.9 (1.0)	1/200 self-harm presentation/year
Consultant 4 have <1 cons/500 beds	1.5 (1–3)	1.6 (0.7)	1.2 (0.3)	7 (1)
Psychology with CLP team	0.6 (0–3)	0.8 (1.1)	0.6 (0.7)	0 (3)
Other psychology	1.8 (0–3)	1.6(1.3)	1.3 (1.0)	4 have no CLP psychology
NCHD	3.0 (1–7)	3.0 (1.9)	2.3 (1.6)	5 have HSTs
HST	1 (0–2)	0.6 (0.7)	0.5 (0.5)	7 had <1/500
BST/SHO/Registrar	1 (0–6)	2.3 (1.6)	1.8 (1.3)	
Other clinical team members	0 (0–1)	0.1 (0.3)	0.1 (0.2)	0 (4)
Administration	1.2 (1–2)	1.4 (0.5)	1.1 (0.4)	2 (2)
**Model 3 hospitals** (***n*** **=** **18**)				
Clinical nurse specialist	2.9 (0–5)	2.5 (1.2)	4.4 (1.8)	5 (7)
Clinical nurse specialist (National clinical programme, self-harm)	1.0 (0–3)	1.1 (0.8)	2.1 (1.5)	1/200 self-harm presentation/year
Consultant	0 (0–1)	0.4 (0.4)	0.6 (0.8)	5
Psychology with CLP team	0 (0–1)	(0.2)	0.1 (0.3)	0 (1 hospital had 2 psychologists; all others 0)
Other psychology	0 (0–2)	0.1 (0.5)	0.3 (1.3)	
NCHD (total)*	0.7 (0–2)	0.7 (0.7)	1.4 (1.7)	3 have HSTs
HST	0 (0–1)	(0.3)	0.2 (0.4)	10 have ≥1 per 500
BST/SHO/Registrar	0.4 (0–2)	0.6 (0.7)	1.3 (1.7)	
Other clinical team members	0 (0)	0 (0)	0 (0)	0 (4)
Administration	0 (0–1)	0.2 (0.3)	0.3 (0.6)	1 (2)
**Paediatric hospitals** (***n*** **=** **2**)				
Clinical nurse specialist	2.5 (2–3)	2.5 (0.7)	7 (3.9)	1 (5) 9.7/500 beds and 4.3/500 beds
Clinical nurse specialist (National clinical programme, self-harm)	1.0 (0–2)	1.0 (1.4)	3.2 (4.6)	1/200 self-harm presentation/year
Consultant	2.2 (2.1–2.2)	2.2 (0.1)	5.8 (1.5)	2 (1)
Psychology with CLP team	1.6 (0–3.2)	1.6 (2.3)	5.2 (7.3)	1 (3) One site has 3 psychologists on CL team, other hospital has 10 non-aligned psychologists
Other psychology	5.5 (1–10)	5.5 (6.4)	12.4 (12.9)	
NCHD (total)^*^	2.3 (2–2.5)	2.3 (0.4)	5.9 (0.8)	2 (1)
HST	(0–0.5)	0.3 (0.4)	0.5 (0.8)	1 service has 0.5 HST
BST/SHO/Registrar	2 (2–2)	2 (0)	5.4 (1.6)	
Other clinical team members	0.5 (0–1)	0.5 (0.7)	1.6 (2.3)	0 (4)

* *NCHD= Non Consultant Hospital Doctor all doctors who are not consultants, including BSTs (Basic Specialist Trainees), HSTs (Higher Specialist Trainees) and SHOs (senior house officers) or registrars (the latter 2 categories may not be in approved training posts)*.

There was a mean of 3.8 WTE (SD 1.3) clinical nurse specialists (CNS) across the hospitals or 2.9 WTE (SD 0.8) nurses/500 beds (including the CNS in self-harm posts from the National Clinical Programme for Self-Harm: NCP: SH), with no hospital reaching the minimum of 5 nursing posts per 500 beds.

There was a mean 0.8 WTE (SD 1.1) psychologist integrated to the liaison psychiatry teams, and 1.6 WTE (SD 1.3) psychology elsewhere in the hospitals. Four hospitals have no psychology available across the hospital as part of the liaison psychiatry service, although there is discrete psychology provision for certain clinical areas.

Four of the Dublin-based Model 4 hospitals have national services such as transplant programmes, oncology programmes and neurosurgery centres which had contributed to resourcing for the transplant psychiatry, neuropsychiatry and psycho-oncology services at these sites. These services are included in the totals, and given the additional needs of these services, create a picture of greater overall resource allocation the is available for the general work of the CLP services.

Across the nine service the mean administration provision was 1.4 WTE (SD 1.1), with only 2 (22%) services meeting the Vision for Change minimum of 2 administrative posts/500 beds.

Of the remaining posts (social work, substance misuse counsellor, occupational therapist, and family therapist) there was a mean 0.1 WTE across the 9 sites with no hospital having the 4 recommended staff members.

Six hospitals (67%) have access to group therapies for at least certain patient groups.

#### Model 3 Hospitals

In Model 3 hospitals mean number of consultants is 0.4 WTE (SD 0.4) and of registrars or junior doctors is 0.7 WTE (SD 0.7). Five of the 18 hospitals (28%) had at least 1 consultant/500 beds, and nine (50%) had at least 1 registrar or junior doctor per 500 beds.

There was a mean of 2.5 WTE (SD 1.2) clinical nurse specialists (CNS) across the hospitals or 4.4 WTE (SD 1.8) nurses/ 500 beds (including the CNS in self-harm posts from the NCP-SH, with 7 hospitals (38.9%) reaching the minimum of 5 WTE nursing posts per 500 beds.

There was a mean 0.1 WTE (SD 0.2) psychologist part of the liaison psychiatry teams, and 0.1 WTE (SD 0.5) psychology elsewhere in the hospitals. Seventeen (94.4%) Model 3 hospitals have no psychology available in the hospital.

Across the eighteen services, the mean administration provision was 0.1 WTE (SD 0.2), with only 4 (22.2%) services meeting the Vision for Change minimum of 2 administrative posts/500 beds. Of the remaining 4 posts across other disciplines recommended by A Vision for Change, there were no posts at any site.

#### Paediatric Hospitals

In the two paediatric hospitals, the mean number of consultants is 2.2 WTE (SD 0.1) and of registrars or junior doctors is 2.3 WTE (SD 0.4). Both hospitals had at least 1 consultant/500 beds, and both had at least 1 registrar or junior doctor per 500 beds.

There was a mean of 2.5 (SD 0.7) WTE clinical nurse specialists (CNS) across the hospitals or 2.9 (SD 0.8) nurses/500 beds, with one hospital reaching the minimum of 5 nursing posts per 500 beds.

There was a mean 1.6 WTE (SD 2.3) psychologists as part of the liaison psychiatry teams, and 5.5 WTE (SD 6.4) psychology elsewhere in the hospitals. Both services met the Vision for Change minimum of 2 administrative posts/500 beds. At time of this survey the remainder of the hospitals nationally where children present have no paediatric liaison psychiatry services, with a minority having inreach from local community CAP services.

### Activity

Not all hospitals were in a position to supply exact activity data, and most provided an approximation. Given that few services have a robust electronic health system, data are mostly collected manually. The data on self-harm was present in the main as the data is central to the operation of the NCP-SH. The data are detailed in Table 2. Based on the mean figures at each hospital type, which are estimates and not complete, we can extrapolate an approximate total of (15,075 Model 4 + 13,266 Model 3 + 1,450 Paediatric) 29,791 ED attendances (8,850 Model 4 + 5,742 Model 3 +240 Paediatric) 14,832 ward based consults, and (2,804 Model 4 + 162 Model 3 + 3,500 Paediatric) 6,466 new clinic appointments. This is an approximate total of 51,089 first contacts in this attendance per annum across the 29 teams. Data on repeat reviews were not available, despite the most complex patients being usually reviewed multiple times on even short admissions (e.g., people with serious self-injury requiring care in critical care, people with complex neuropsychiatric conditions, or with serious sequelae of treatment, such as steroid-induced psychosis).

### Care Delivery

All Model 4 hospitals provide a services to the ED and to the medical and surgical wards. Eight (89%) Model 4 hospital CLP services have outpatient clinics too, as do both paediatric CLP services. Only two (11%) of Model 3 hospitals have outpatient CLP clinics. A majority of services provide assessment in the ED in parallel with medical treatments where indicated, which is consistent with best practise (15).

None of the Dublin based hospitals have co-located maternity services—in the capital these are located at 3 standalone maternity hospitals. All other Model 4 hospitals (*n* = 4) have obstetric services on site, three are designated “hubs” for perinatal mental health care (have a perinatal mental health team on site), and one is a “spoke” as designated by the Perinatal Model of Care (16). Twelve of the Model 3 hospitals are designated “spokes” and have mental health midwives who can escalate mental health need to the consultant liaison psychiatrist.

### Specialist Services for Different Age Groups

In Dublin, there are 2 paediatric hospitals which see all patients under 16 years of age. In Dublin, those aged 16 and over who require acute medical care are seen in the EDs of adult hospitals. There is no expertise in Child and Adolescent Psychiatry (CAP) at any of the Dublin EDs, and care is provided by the adult services both within and outside working hours.

In other parts of the country, the ED caters for all age groups. Outside of Dublin there is one paediatric liaison psychiatry service in Cork, which provides a service to a Model 4 and a Model 3 hospital. There is variable provision of CAP expertise in the remaining Model 3 and Model 4 hospitals.

Three of the Dublin hospitals have bespoke Psychiatry of Old Age (POA) staffing. In one hospital this is closely integrated with the working age service and together provides a single point of access. The other two have different referral pathways. In the remaining hospitals there is a varying degree of “inreach” provided by the POA teams in the local area, and in the main they only provide a service to patients who meet the criteria for their community service—thus tertiary or out of area patients have mental health care provided by the non-specialist working-age service. This practise is supported by the Model of Care for Older People (17).

### Funding and Governance

Of the 9 Model 4 Hospitals, 6 (67%) CLP services are funded principally by the acute hospital, with the exception of the CNSs from the NCP-SH, which are funded through the mental health budget. Of the four non-Dublin based Model 4 hospitals, three have CLP services funded via the mental health services, with a hybrid model in one. The CLP services in Model 3 hospitals are funded by the mental health services with the exception of two hospitals where there is a hybrid model in place. The two paediatric hospitals fund their CLP services.

## Discussion

This study found that Irish CLP services are not resourced to the level recommended by the 2006 policy document A Vision for Change. When a benchmarking exercise was used to compare staffing levels per 500 beds with the standard, no services were adequately resourced.

The Model 4 or tertiary hospitals were significantly bigger and busier than the Model 3 hospitals, and their work is more of the complex mix of CLP rather than the predominance of emergency psychiatry at the smaller sites. All services are severely under-resourced by any defined standards. Model 4 hospitals had levels of medical and psychological provision which were on the surface at the levels set out by A Vision for Change. More specialised services require higher levels of medical input due to the level of complexity of the patient group. They do not have the levels of nursing staff required to safely provide 9–5 level staffing, much less any extended hours offering. Notwithstanding these deficits, 33% of services provided some form of extended hours services, although none provided a 24 h service.

When benchmarked against the international standards of the NHS England-NICE standards in England and Wales, no adult services meet the level of resourcing for CORE services, much less CORE-24, as no services ran 24/7 (13). Like in Norway, the availability of CLP services outside of working hours is severely limited in this study (18). However, consistent with many services in England, several Model 4 hospitals had elements of Comprehensive services (i.e., highly specialised input for discrete clinical areas, e.g., neuropsychiatry, psycho-oncology, transplant psychiatry), combined with “sub-Core” provision across the service more generally. Existing services have developed organically at certain major national centres by individual funding by these tertiary/national services at these hospitals. These were similar to “Cluster 3” services identified in the UK by Walker et al. (10). Unlike services in hospitals on continental Europe there is little history of psychosomatic medicine services. Compared with a European survey of CLP services published 20 years ago, the Irish Model 4 hospitals are equivalent to Cluster II and III and the Model 3 to Cluster I, and found a mix of traditional medical model and multidisciplinary services (19). This study, however was an opt-in model and likely included those services who were most interested in research and service development. A more recent Italian study of 5 hospitals of various sizes concluded that the better resources and more active a hospital service, the more likely patients were to be referred, and that the existence of strong services overcame systemic barriers to patients receiving needed mental health care, consistent with finding of Chen et al. from an Australian study (20, 21). Lobo et al. in a survey of Spanish CLP services reported higher proportions of services with psychology provision and less specialist nursing provision. Neither this Spanish study, nor a similar Norwegian described the degree of ED input (18, 22).

The existing levels of nursing resourcing owe much to the NCP-SH which has set the standard for the staffing of mental health services at EDs at 1 CNS/200 presentations per annum, and without which the mean CNS staffing levels would certainly be much lower especially in the Model 3 hospitals (2, 23). This, along with the effective delivery of the Perinatal Model of Care, emphasise the importance of funding accompanying staffing requirements to ensure services are adequately resourced (16).

Among Model 3 hospitals, there were higher staffing levels for nursing staff compared with Model 4 hospitals (39% meeting the standard of 5 CNS/500 beds, compared with none in the Model 4 hospitals), but this is counterbalanced by the lack of medical staff. The five hospitals with consultant staffing (any WTE) met the minimum standard of A Vision for Change. While it may be difficult to justify a fulltime CLP consultant for a hospital with 200 beds, this indicates a need for some degree of on-site medical leadership and there may be potential to combine a 0.5 WTE CLP consultant role with other roles in psychiatry or to provide part-time working options.

For the paediatric hospitals, there were no staffing levels specified in A Vision for Change and UK-based documents did not specify what resourcing of paediatric hospitals should be. However, as the assessment and treatment of children and adolescents is arguably a lengthier process in the main, there is an argument for higher levels of staffing per presentation in these settings (24).

Resourcing levels in Ireland compare poorly to English levels of staffing and service delivery. With all services being sub-core, the resources are not in place to provide high quality 24/7 care. In the UK, 21% met at least Core standards, and 10.1% were at least Core24 (10). Activity levels are higher than those reported by services in UK and Australia although the levels of resourcing are poor (25, 26). There is an identified need to ensure services are resourced to the minimum levels established by national and international standards.

Data and information systems are a significant barrier to the development of services. The provision of these is idiosyncratic and there is no national standard. Where hospitals have data and information systems in place it allows for a more granular examination of the work of the CLP service (27, 28). Universal information systems would allow comparisons to be made to similar work conducted internationally, and to establish whether the work of Irish CLP differs from other countries, and where it needs to be improved (25, 29, 30). The data available suggests very high levels of clinical activity, compared to other areas of mental health services.

## Limitations

The most significant limitation was the absence of any systematic consistent activity data across the sites, as a result this study was dependent on self-report data, rather than independent observation. However, this is not dissimilar from other published work in the area of CLP service-based research (10). At the smaller sites where self-harm data represents a significant proportion of the workload, this is captured by the NCP-SH data. It is difficult for clinical teams with little administrative support, often struggling to meet the clinical demands for their services, to prioritise data collection ahead of delivering clinical care.

The full (100%) response rate gives a clear view of the national provision of services, similar to the work of Walker et al. in the UK (10). It is a very strong response compared with the 62% response rate in the Spanish study and the 41% response rate in a similar Norwegian study (18, 22).

The findings of this study will be used as a basis of a Model of Care for Irish CLP services, a model that will be developed in keeping with the recommendations of the 2020 policy document Sharing the Vision (31). It is expected that this will result in the implementation of minimum safe resourcing levels and allocation of information systems and administration to facilitate future development of Irish CLP services.

Further work will incorporate the patient voice into both the design as well as capturing the qualitative experience of patients who come into clinical contact with liaison psychiatry services. There is a clear need for robust data to be routinely collected in order to identify the areas where there is greatest need as well as simply quantifying activity. This is a challenge internationally (32). Such data would allow for the activity-based commissioning of services and would lead the way toward ensuring that services are adequately resourced for the needs of their patients. Once there is data available it will be possible to establish outcome measurements such as the FROM-LP and to use this to enhance the quality of services delivered (33).

## Conclusions

This study found that despite the strong and evolving data on the economic benefits of a well-functioning CLP service the development of CLP services in Ireland is lagging behind the development of policy. Despite this, services provide high volume of care, and a high proportion are adhering to best practise guidelines such as regarding parallel assessment. In Ireland, CLP services are grossly under-resourced, and report high levels of clinical activity. There is an urgent need for robust data collection and for investment to bring these services to a sustainable level of resourcing for the activity undertaken. This paper has demonstrated the need for a systematic approach to developing and evaluating services, with twin key areas: adequate staffing and information systems to capture data on service need and activity.

## Data Availability Statement

The raw data supporting the conclusions of this article will be made available by the authors, without undue reservation.

## Author Contributions

AD, MC, EK, EC, EB, EG, and SM conceived of the research. WL provided external advice. RP, KM, and AD carried out the survey. AD carried out the statistical analysis and wrote the first draught. All authors contributed to the manuscript and approved the final version.

## Conflict of Interest

The authors declare that the research was conducted in the absence of any commercial or financial relationships that could be construed as a potential conflict of interest.

## Publisher's Note

All claims expressed in this article are solely those of the authors and do not necessarily represent those of their affiliated organizations, or those of the publisher, the editors and the reviewers. Any product that may be evaluated in this article, or claim that may be made by its manufacturer, is not guaranteed or endorsed by the publisher.
