# Supplementary material for: Consultation-Liaison Psychiatry Services in Ireland: A National Cross-Sectional Study
Source: Front Psychiatry. 2021 Nov 29;12:748224. doi: 10.3389/fpsyt.2021.748224 (PMC8666631; doi:10.3389/fpsyt.2021.748224)
Supplement: Supplementary file 1 [file Data_Sheet_1.pdf]

Liaison Psychiatry Survey of Ireland

This is the First Liaison Psychiatry Survey of Ireland. In this survey we will ask some questions about your service - its level of resourcing and activity levels. We hope to use this information to inform future service development in the Faculty model of care, to identify areas of need, and also areas of excellence from which national services can learn.

If you know your service's activity levels this will help in the relevant section.

These questions have been based around the scenario in most Model 3/4 hospitals with an emergency department (ED). Some questions may not apply to you, e.g if your service is in a paediatric hospital.

All responses will be read by a human, so please just describe your situation if the questions don't adequately capture the detail of your service. There will be free text sections throughout, and especially at the end.

* Required

Staffing, resources and service delivery

1. Name of hospital *

____

2. What is your clinical role? *

Mark only one oval.

- ◯ Consultant in Adult Liaison Psychiatry
- ◯ Consultant in Child and Adolescent Liaison Psychiatry
- ◯ Consultant in Liaison Psychiatry of Later Life
- ◯ Consultant in Subspecialist Liaison Psychiatry (psycho-oncology, transplant psychiatry, perinatal)
- ◯ Other Consultant Psychiatrist
- ◯ Clinical Nurse Specialist
- ◯ Advanced Nurse Practitioner
- ◯ Other: ____________________

3. If other please expand

4. Regarding your services, please select all that apply *

Check all that apply.

- [ ] Hospital has an ED
- [ ] Liaison psychiatry service provides assessments in the ED
- [ ] Liaison psychiatry service provides assessments to acute medical wards
- [ ] Liaison psychiatry provides a dedicated OPD service
- [ ] Other teams provide mental health services to your hospital (e.g psychiatry of old age, CAMHS etc) - please describe

Other: [ ] ______

5. If other teams provide MHS to your hospital please describe

6. No of beds in acute hospital (approx) *

7. Number of Consultants (WTE: Whole Time Equivalent e.g. 0.6 if one 3 days/week) *

8. Number of HSTs (WTE) *

9. Number of BSTs dedicated to the liaison psychiatry team (WTE) *

10. Number of other doctors, i.e. SHOs/non-HST Registrars/GP trainees (WTE) *

11. Number of nurses (WTE) *

12. How many of the nurses (WTE) are from the National Clinical Programme for Self Harm(SH)? Do they see non SH patients? *

13. Number of admin staff (WTE) *

14. Number of psychologists on liaison psychiatry team (WTE) *

15. Any other disciplines dedicated to liaison psychiatry: OT, SW, physio etc (WTE) *

16. Are there other psychologists in the hospital, not part of the liaison psychiatry team? Please describe areas & WTE. *

17. Are there group therapies or self-management groups available to patients in your hospital? Please briefly describe

18. Are there any subspecialties or special interest areas which have received specific funding (e.g. for oncology, transplant, neuropsych, gender etc)? Please describe: *

19. Are there any unfilled posts in your service? e.g. a consultant post advertised but unfilled. Please describe.

20. Is there specialist input from psychiatry of later life? *

Mark only one oval.

- All >65y
- >65y from catchment area
- No psychiatry of later life
- Other: ______________________

21. If 'other', please describe...

____
____
____
____
____

22. Is there specialist input from child and adolescent psychiatry (if not a paediatric hospital)? *

Mark only one oval.

- All <16y
- All <18
- No CAMHS input at all
- Other: ______________________

23. If your hospital has an obstetric unit, do you have a perinatal team? *

Mark only one oval.

- No obstetric unit
- Obstetric unit - a perinatal hub with dedicated Perinatal Psychiatry
- Obstetric unit - a perinatal 'spoke' with a mental health midwife
- Obstetric unit - no dedicated staffing
- Other: ______________________

24. Please add any additional information here:

____

Activity

if possible, could you answer 5 brief questions on activity please?

25. How many ED attendances/year (approx)?

____

26. Regarding ED assessments please tick all that apply: *

Check all that apply.

- [ ] Assess patients referred following Emergency Medicine review
- [ ] Assess patients in parallel with Emergency Medicine
- [ ] Assess patients directly from Triage
- [ ] Other

27. If other, please describe

____

28. How many new referrals/year from hospital wards?

29. How many attendances with self-harm/year?

30. Can you estimate outpatient activity (new/return)?

31. Please add any additional information here:

Resourcing

32. How is your team funded? Please tick all that apply *

Check all that apply.

- [ ] Acute hospital budget, HSE hospital
- [ ] Acute hospital budget, non-HSE hospital
- [ ] Mental health/CHO budget
- [ ] National Clinical Care Program
- [ ] Other

33. If other please expand:

34. What (if any) non-liaison psychiatry emergency resources are available in your area? *

Check all that apply.

- [ ] Crisis Team
- [ ] Direct access to urgent CMHT review
- [ ] Other
- [ ] None

35. If other please expand:

36. What are the hours of your service? i.e. 8-5, 9-5, 8-8 etc *

37. Do you have adequate office space? *

Mark only one oval.

- ( ) Yes
- ( ) No

38. Is your office in the acute hospital? *

Mark only one oval.

- Yes
- No

39. Are there any problems with your office space? Please briefly outline

40. Is there any other information about activity and resourcing that you would like to add.

41. What do you feel your service does well?

42. What are the main challenges for your service?

43. Is there anything else you would like to contribute to this survey? (Perhaps your service has piloted an initiative, or accessed specific funding eg. for Covid-19 - please tell us the specifics of your unique service)

44. If you would be happy to be contacted, please leave your contact details (name, phone no, email)

Many thanks for taking the time to complete this survey.

This content is neither created nor endorsed by Google.

Google Forms
